# Two-step interphase microtubule disassembly aids spindle morphogenesis

**DOI:** 10.1186/s12915-017-0478-z

**Published:** 2018-01-23

**Authors:** Nunu Mchedlishvili, Helen K. Matthews, Adam Corrigan, Buzz Baum

**Affiliations:** MRC Laboratory of Molecular Cell Biology and the IPLS, University College London, Gower Street, London, WC1E 6BT UK

**Keywords:** Mitosis, Spindle assembly, Nuclear envelope breakdown, Ensconsin/MAP7, Microtubules Multipolar spindle

## Abstract

**Background:**

Entry into mitosis triggers profound changes in cell shape and cytoskeletal organisation. Here, by studying microtubule remodelling in human flat mitotic cells, we identify a two-step process of interphase microtubule disassembly.

**Results:**

First, a microtubule-stabilising protein, Ensconsin/MAP7, is inactivated in prophase as a consequence of its phosphorylation downstream of Cdk1/cyclin B. This leads to a reduction in interphase microtubule stability that may help to fuel the growth of centrosomally nucleated microtubules. The peripheral interphase microtubules that remain are then rapidly lost as the concentration of tubulin heterodimers falls following dissolution of the nuclear compartment boundary. Finally, we show that a failure to destabilise microtubules in prophase leads to the formation of microtubule clumps, which interfere with spindle assembly.

**Conclusions:**

This analysis highlights the importance of the step-wise remodelling of the microtubule cytoskeleton and the significance of permeabilisation of the nuclear envelope in coordinating the changes in cellular organisation and biochemistry that accompany mitotic entry.

**Electronic supplementary material:**

The online version of this article 10.1186/s12915-017-0478-z) contains supplementary material, which is available to authorized users.

## Background

The goal of mitosis is the equal segregation of genetic material into two daughter cells. To achieve this, animal cells undergo profound changes in cell organisation. Cells round up, chromosomes condense and the permeability of the nuclear envelope increases (a process we term nuclear envelope permeabilisation (NEP)), leading to mixing of nucleoplasm and cytoplasm. At the same time, the array of long interphase microtubules is replaced by a population of short and highly dynamic centrosomally nucleated microtubules, which go on to form the mitotic spindle — the structure responsible for chromosome segregation.

Microtubule cytoskeleton remodelling starts before NEP with a dramatic increase in microtubule nucleation at the centrosomes [1], driven by the recruitment and local activation of the gamma tubulin ring complex (γ − TuRC) [2–5]. While this burst of centrosomal microtubule nucleation is easily visible in most cell types, careful quantification of microtubule polymer levels has suggested that total cellular levels of the tubulin polymer remain relatively constant during this period, before abruptly dropping at the transition from prophase to prometaphase with NEP [6]. Once cells are in prometaphase, highly dynamic microtubules emanating from the centrosomes search cell space for kinetochore attachment sites on which to capture chromosomes [7–9]. This stabilises the microtubules, leading to a rise in tubulin polymer levels during mitotic spindle formation [6, 10]. Close to the chromosomes, microtubule polymerisation is aided by a local Ran-guanosine triphosphate (GTP) gradient [11–17]. The final size of the spindle is then determined by microtubule regulators [18] together with the total available pool of tubulin heterodimers [19].

The wholesale change in cell state at mitotic entry is driven by the activation of Cdk1/cyclin B complex — the master regulator of mitosis [20]. Förster resonance energy transfer (FRET)-based quantitative measurements in mammalian somatic cells revealed that Cdk1/cyclin B complex first becomes activated in the cytoplasm around 20 min before NEP [21]. The activity of Cdk1/cyclin B then increases through the action of positive feedback loops [22], peaking around 10 min after the onset of prometaphase [21]. This change in Cdk1/cyclin B activity is accompanied by a change in its localisation. At early stages the complex is seen at elevated levels in the cytoplasm, where it is concentrated at the centrosomes in many systems [23, 24]. Then, as levels of Cdk1/cyclin B increase, the complex enters into the nucleus [25], where it triggers the onset of NEP [26]. Once the nuclear envelope has been permeabilised, cells are committed to mitosis [27].

It has generally been assumed that the differences in microtubule structure and dynamics that accompany the transition from interphase to mitosis result from phosphorylation-induced changes in the activities and/or binding capacities of stabilising and destabilising microtubule-associated proteins (MAPs) downstream of Cdk1/cyclin B activation [28–32]. However, the rise in Cdk1/cyclin B activity in prophase that precedes and drives mitotic entry [21] does not mirror the dynamic changes in overall microtubule polymer levels, which occur over a very short period at the transition from prophase to prometaphase [6]. This suggests that there is more at play. Thus, in this paper, we set out to shed new light on the little-understood process of interphase microtubule remodelling as cells enter mitosis.

First, to overcome the difficulties of studying microtubules in cells undergoing mitotic rounding, we carried out a detailed quantitative analysis of microtubule polymer levels in flat cells, where the microtubules can be easily visualised. Through this analysis, interphase microtubule disassembly was found to occur in two sequential steps. During prophase, when levels of mitotic kinase activity rise, interphase microtubules were gradually disassembled, in part as a result of phosphorylation-induced inactivation of the microtubule stabiliser Ensconsin/MAP7. Subsequently, the remaining interphase microtubules were rapidly lost as the concentration of tubulin heterodimers fell during permeabilisation of the nuclear envelope. Finally, a failure to inactivate Ensconsin/MAP7 prior to NEP was found to lead to the persistence of clumps of interphase microtubules through into metaphase — making clear the importance of microtubule remodelling being a multistep process.

## Results

### The interphase microtubule cytoskeleton is remodelled in two discrete steps at mitotic entry

**Fig. 1:**
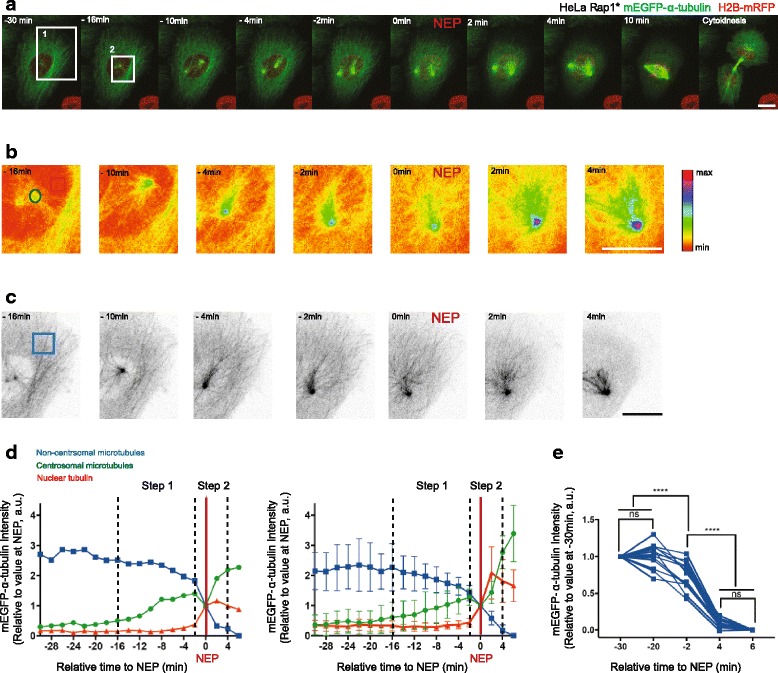
Disassembly of interphase microtubules begins prior to NEP and is accelerated at NEP. **a** Representative time-lapse confocal images (*x*-*y* maximum projection) of a HeLa cell stably expressing H2B-mRFP (to visualise chromosomes) and mEGFP-α-tubulin (to visualise microtubules and NEP) and transiently overexpressing Rap1* (to keep cell flat in mitosis). Boxed areas show regions zoomed in **b** and **c. b** Higher magnification (sum projection of mEGFP-α-tubulin sections around the centrosome, pseudo-color, spectra look-up table (LUT)) of boxed region 2 indicated in **a** showing changes of mEGFP-α-tubulin levels at the centrosome relative to NEP. Insets indicate regions used for quantifications: *green* (centrosomal microtubules), *red* (nuclear tubulin). **c** Higher magnification (maximum projection of mEGFP-α-tubulin basal sections, inverted greyscale) of region 1 in **a** showing that non-centrosomal microtubule disassembly is triggered before NEP and accelerates during loss of the nuclear-cytoplasmic compartment boundary. Boxed area indicates region used for quantifications. **d** Changes in median centrosomal and non-centrosomal microtubule intensity relative to NEP for H2B-mRFP mEGFP-α-tubulin HeLa cell transiently overexpressing Rap1* (shown in **a**–**c**, *left*) and for five equivalent cells from two independent experiments (*right*). Median intensity of mEGFP-α-tubulin signal was calculated within a 15 × 15 pixel circle around the centrosome (*green line*, as indicated in **b**), a 10 × 10 pixel box within the nucleus (*red*, as indicated in **b**) and a 30 × 30 pixel box at the cell periphery (*blue*, as indicated in **c**). Time point 0 represents NEP. Graph shows mean and standard deviation (SD). **e** Changes in non-centrosomal microtubule levels relative to NEP. Measurements show median of mEGFP-α-tubulin signal in a 30 × 30 pixel box at two locations at the periphery of a cell as shown in Additional file 1A at –30, –20, –2 and 4, 6 min relative to NEP in H2B-mRFP mEGFP-α-tubulin HeLa stable cell line transiently overexpressing Rap1* (13 cells (including 5 cells from **d**), four independent experiments). Repeated measures analysis of variance (ANOVA), Tukey's multiple comparisons test with a single pooled variance, ****P < 0.0001. Scale bars represent 10 μm

In order to explore the kinetics of microtubule remodelling upon entry into mitosis, we began by monitoring changes in the microtubule cytoskeleton organisation relative to loss of the nuclear-cytoplasmic compartment boundary (NEP) in HeLa cells stably expressing histone-2B-monomeric red fluorescent protein (mRFP) and monomeric enhanced green fluorescent protein (mEGFP)-α-tubulin [33]. To better visualise microtubule remodelling during this period, cells were transfected with a constitutively activated version of the small GTPase Rap1 (here called Rap1*), which prevents focal adhesion disassembly [34, 35] (Fig. 1a, Additional file 1A). Several processes were observed. As previously described [36], there was a steady accumulation of mEGFP-α-tubulin at the centrosomes in prophase (Fig. 1a, b), which was accompanied by the gradual disassembly of peripherally localised interphase microtubules (Fig. 1c). This continued up until NEP, when dramatic and sudden loss of microtubule polymers occurred over the course of a few minutes (Fig. 1a–c).

To gain a quantitative measure of the changes in microtubule polymer levels that accompany entry into mitosis, we followed changes in the intensity of the mEGFP-α-tubulin polymer signal at the centrosomes, using the rise in nuclear mEGFP-α-tubulin as a marker of NEP (Fig. 1d, Additional file 1 F) and mEGFP-α-tubulin in neighbouring cells to control for bleaching (Additional file 1B–D). At the same time, we used mEGFP-α-tubulin to determine the kinetics of interphase microtubule disassembly at the cell periphery (beyond the reach of visible microtubules emanating from the centrosome) (Fig. 1a, c, d, e). Since this population of interphase microtubules is not physically connected to the centrosome in prophase, we refer to them here as “non-centrosomal microtubules”. In line with our qualitative observations, the increase in the mEGFP-α-tubulin signal at the centrosomes began ~ 16 min before NEP (Fig. 1d, Additional file 1 F) and was accompanied by a steady decrease in levels of mEGFP-α-tubulin polymer (a 33 ± 17% decrease in mEGFP-α-tubulin intensity over 14 min) at the cell periphery (Fig. 1d). This was confirmed using the local variance of mEGFP-α-tubulin intensity as an alternative method by which to quantify levels of tubulin polymer above the background monomer signal (Additional file 1E). Strikingly, these gradual changes in the levels of tubulin polymer during prophase were abruptly altered at NEP, when the sudden increase in nuclear mEGFP-α-tubulin was accompanied by a transient reduction in the levels of microtubule polymer at the centrosomes and the rapid loss of residual microtubules from the cell periphery (Fig. 1a–e, Additional file 1 F).

These results, in line with those of previous studies [6], suggest that the remodelling of the microtubule cytoskeleton at mitotic entry involves discrete processes. The first step, during prophase, is characterised by a slow partial depolymerisation of interphase microtubules at the cell periphery and the nucleation of microtubules from centrosomes. Since these processes occur in parallel, total levels of tubulin polymer do not markedly change during this period [6]. Subsequently, coincident with the loss of the nuclear-cytoplasmic compartment barrier, there is a sudden reduction in the total levels of microtubule polymer (Fig. 1a–c).

### Step 1: Cdk1/cyclin B-dependent removal of Ensconsin/MAP7 from microtubules triggers non-centrosomal microtubule depolymerisation during prophase

**Fig. 2:**
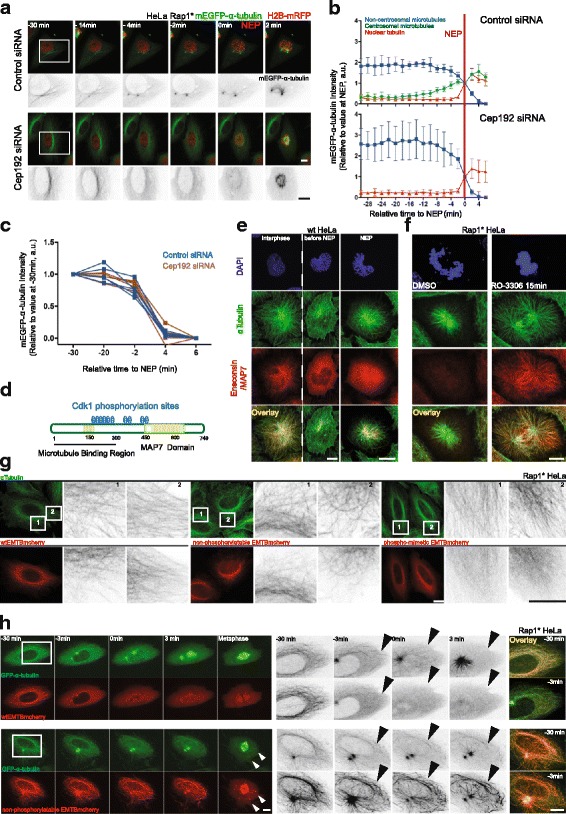
Removal of Ensconsin/MAP7 by Cdk1/cyclin B activation triggers non-centrosomal microtubule depolymerisation before NEP. **a** Representative time-lapse confocal images (*x*-*y* maximum projection) of HeLa cells during mitotic entry stably expressing H2B-mRFP and mEGFP-α-tubulin and transiently overexpressing Rap1* treated with control small interfering RNA (siRNA) (*upper panel*) or Cep192 siRNA (*lower panel*). **b** Changes in centrosomal and non-centrosomal microtubule levels relative to NEP for control siRNA (*upper panel*, five cells) and for Cep192 siRNA (*lower panel*, five cells) (see Methods for measurement details). Two independent experiments. Graphs show mean and SD. **c** Changes in non-centrosomal microtubule levels relative to NEP for cells in **b**. Median of α-tubulin-GFP signal was measured as described in Fig. 1e. **d** Schematic representation of Ensconsin/MAP7 structure. **e** Representative confocal images (*x*-*y* maximum projection) of fixed HeLa cells stained to show that Ensconsin/MAP7 is removed from microtubules in prophase. Ensconsin/MAP7 in *red*, α-tubulin in *green* and 4’,6-diamidino-2-phenylindole (*DAPI*) in *blue* (11 prophase, > 30 interphase cells, two independent experiments). **f** Representative confocal images of fixed HeLa cells overexpressing Rap1* stained to show that Ensconsin/MAP7 relocalises to the microtubules upon Cdk1 inhibition with RO-3306. Ensconsin/MAP7 in *red*, α-tubulin in *green* and DAPI in *blue* (9 dimethyl sulfoxide (*DMSO*) and 10 RO-3306 cells, two independent experiments). **g** Representative confocal images of fixed HeLa cells overexpressing Rap1* stained to show that overexpressed Wt-EMTB-mCherry (9 cells) and a non-phosphorylatable mutant (A-EMTB-mCherry, 14 cells) remain bound to microtubules in interphase, whereas the phospho-mimetic (E-EMTB-mCherry, 10 cells) remains largely cytoplasmic. α-Tubulin in *green*, mCherry in *red* (one experiment). **h** Representative time-lapse confocal images (*x*-*y* maximum projection) of flat (Rap1*) HeLa cells stably expressing GFP-α-tubulin and Wt-EMTBmCherry (*upper panel*, 18 cells) or its non-phosphorylatable mutant (A-EMTB-mCherry, *lower panel*, 15 cells) to show that the A-EMTB-mCherry stays associated with microtubules during prophase, leading to delay in microtubule disassembly at mitotic entry. *White arrows* indicate microtubule clumps. *Black arrows* indicate interphase microtubules just before or after NEP (five independent experiments). Boxed areas show regions zoomed in inverted greyscale. Scale bars represent 10 μm

To elucidate the molecular mechanisms that govern microtubule disassembly at the entry of mitosis, we focused first on events during prophase. In order to test whether the loss of interphase microtubules during this period is an indirect consequence of the growth of centrosomal microtubules, e.g. via competition for a common tubulin heterodimer pool, we monitored the loss of interphase microtubules in cells that fail to nucleate centrosomal microtubules as the result of RNA interference (RNAi)-mediated silencing of Cep192 [37, 38] (Fig. 2a). The RNAi had the expected effects [37, 38]. Importantly, however, Cep192 silencing did not significantly alter the disassembly kinetics of peripheral interphase microtubules (Fig. 2a–c, Additional file 2A), implying that the two processes, interphase microtubule loss and centrosome maturation, are regulated independently of one another.

Cdk1/cyclin B complex activity rises at around the time that interphase microtubules begin to depolymerise [26]. Since Cdk1/cyclin B is thought to mediate many of the structural changes that accompany entry into mitosis, including microtubule cytoskeleton remodelling [28–32], in our search for regulators of this loss of interphase microtubules at the entry into mitosis, we focused our attention on conserved regulators of microtubules that are potential substrates of Cdk1/cyclin B. This led us to explore the function of Ensconsin/MAP7 in this system. This choice was based upon the fact that Ensconsin (also called MAP-7 and E-MAP-115) (Fig. 2d) is a microtubule-associated protein that is phosphorylated in mitosis [39]. Moreover, while both fly and human Ensconsin/MAP7 homologues function to stabilise microtubules [40–42], the hyper-phosphorylated, mitotic form of the protein is unable to bind to microtubules in vitro and in HeLa cells [39]. Furthermore, a quantitative proteomics study identified Ensconsin/MAP7 as a protein that preferentially binds interphase microtubules [43]. Although data from some cells types do not entirely fit with this model [44], taken together this previous work suggests Ensconsin/MAP7 as a regulator of changes in microtubule dynamics during passage from interphase into mitosis.

To begin our analysis of the localisation, regulation and function of Ensconsin/MAP7, we used immunofluorescence to visualise changes in the localisation of the endogenous protein during mitotic entry (Fig. 2e). The specificity of the antibody was confirmed via western blot using extracts prepared from cells treated with three different small interfering RNAs (siRNAs) targeting Ensconsin/MAP7 mRNA and by immunofluorescence (Additional file 2B, C). As previously reported [39], Ensconsin/MAP7 decorated interphase microtubules in untreated HeLa cells but was lost from microtubules in early prophase (Fig. 2e). The same result was obtained in MCF10A cells (Additional file 2D). Since the Ensconsin/MAP7 microtubule-binding domain (EMTB) was previously shown to have the same subcellular localisation as the full-length protein [45] and to be sufficient to stabilise microtubules against drug-induced disassembly in vivo [40], we used this shorter version of the protein to dissect the mechanisms controlling the change in its localisation.

Like the endogenous protein, when EMTB-mCherry was expressed in flat Rap1* HeLa cells stably expressing GFP-α-tubulin [46], it localised to microtubules in interphase (Fig. 2h, upper panel, Additional file 2 F, upper panel), but it was lost from microtubules as cells entered mitosis (Fig. 2h, upper panel, Additional file 2 F, upper panel) — a result that was confirmed in fixed cells (Fig. 2g, Additional file 2E, upper panel). Significantly, the dissociation of EMTB from interphase microtubules was found to precede their depolymerisation (Fig. 2h, upper panel, Additional file 2 F upper panel, Additional file 2E, upper panel).

To determine if the removal of Ensconsin/MAP7 from microtubules at the onset of mitosis was sensitive to changes in the levels of the Cdk1/cyclin B kinase activity (Fig. 2d), we treated HeLa cells overexpressing Rap1* with the Cdk1 inhibitor RO-3306 (or dimethyl sulfoxide (DMSO)) for 15 min (Fig. 2f). Cells were then fixed and stained for α-tubulin, Ensconsin/MAP7 and DNA. In cells forced to exit mitosis as the result of treatment with Cdk1 inhibitor (Fig. 2f), there was a visible increase in microtubules, which were associated with Ensconsin/MAP7. In addition, to determine whether the Cdk1/cyclin B-dependent dissociation of Ensconsin/MAP7 from microtubules is mediated by mitotic phospho-regulation, we manually searched the protein sequence for kinase consensus sites (Fig. 2d). This identified ten Cdk1 sites (T/SP) in the protein, six of which were present in the microtubule-binding domain of Ensconsin/MAP7, which we showed mimics the behaviour of the full-length protein. All six sites were previously identified as sites of phosphorylation in mitotic cells using mass spectrometry [47, 48] (just one of these sites was found to be phosphorylated in both mitosis and G1 [47]), together with four potential Nek2 sites in the region. To determine the function of the ten putative mitotic kinase sites, we generated non-phosphorylatable (here called A-EMTB-mCherry) and phospho-mimetic variants of EMTB-mCherry (here called E-EMTB-mCherry). These constructs were then transiently expressed in HeLa cells, and the cells were fixed and labelled with anti-α-tubulin and anti-mCherry antibodies. Whereas both the wild-type and the A-EMTB-mCherry constructs decorated interphase microtubules, the E-mutant EMTB protein remained diffuse in the cytoplasm (Fig. 2g). Furthermore, unlike the wild-type EMTB-mCherry construct, the A-mutant EMTB-mCherry protein remained tightly associated with microtubules in prophase (Additional file 2E). Together, these data support the hypothesis that Ensconsin/MAP7’s association with microtubules at the onset of mitosis is regulated by phosphorylation within the microtubule-binding domain.

To test whether this phospho-regulation has an impact on interphase microtubule disassembly, we transfected a HeLa GFP-α-tubulin stable cell line expressing Rap1* with either a wild-type or an A-mutant version of the EMTB-mCherry construct and monitored the remodelling of the microtubule cytoskeleton as the cells entered mitosis. Strikingly, the continued association of the A-mutant EMTB-mCherry protein with interphase microtubules was sufficient to increase their stability, so that many now formed clumps in prophase and persisted into prometaphase (Fig. 2h, Additional file 2 F, Additional file 3). These microtubule clusters then became incorporated into the developing spindle or were maintained intact outside the spindle until the end of division (Additional file 3). Thus, the phosphorylation of Ensconsin/MAP7 within the microtubule-binding domain is important for the timely disassembly of interphase microtubules during mitotic entry.

### The function of interphase microtubule disassembly prior to loss of the nuclear-cytoplasmic compartment boundary

**Fig. 3:**
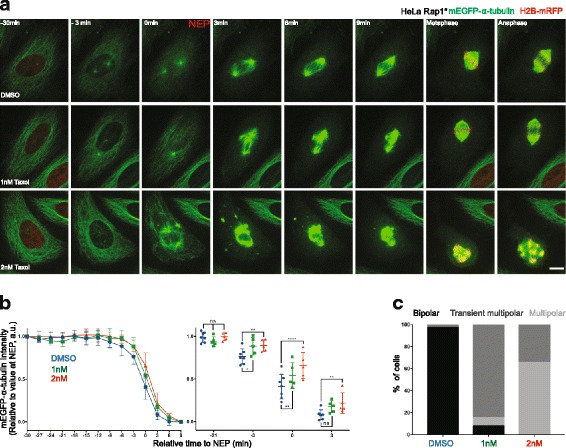
Destabilisation of non-centrosomal microtubules prior to NEP is important for normal spindle assembly. **a** Representative time-lapse confocal images (*x*-*y* maximum projection) of a flat (Rap1*) HeLa cell during mitotic progression stably expressing mEGFP-α-tubulin and H2B-mRFP (shown only at –30, during metaphase and anaphase, to better visualise microtubules), treated with DMSO (*upper panel*), 1 nM Taxol (*middle panel*) or 2 nM Taxol (*lower panel*). **b** Changes in non-centrosomal microtubule levels relative to NEP for cells treated with DMSO (*blue*, seven cells pooled, two independent experiments), 2 nM Taxol (*red*, five cells, one independent experiment) or 1 nM Taxol (*green*, five cells, one independent experiment). Measurements are performed as described in Fig. 1e. Graphs show mean and SD. *Right panel*: comparison between DMSO and 1 nM and 2nM Taxol at –21, –3, 0 and 3 min relative to NEP. Repeated measures two-way ANOVA, Dunnett's multiple comparisons test, ****P ≤ 0.0001, **P ≤ 0.01, *P ≤ 0.05. **c** Quantification of percent mitotic spindle defects in cells (as above) treated with DMSO (34 cells, four independent experiments), 1 nM Taxol (13 cells, two independent experiments) or 2 nM Taxol (26 cells, two independent experiments). Scale bar represents 10 μm

Interestingly, similar microtubule clumps have been previously described in mitotic cells treated with the microtubule-stabilising drug Taxol (paclitaxel) [49–51]. Therefore, to test whether Taxol-induced microtubule clumps have a similar aetiology, we treated cells with 2 nM Taxol, a dose previously shown to stabilise microtubules [52]. This was sufficient to prevent the interphase microtubule disassembly during prophase (Fig. 3a, b), leading to the formation of stable microtubule clumps, many of which failed to resolve prior to spindle formation. Moreover, in some instances, this led to the formation of multipolar spindles, which underwent multipolar divisions in both flat (Fig. 3a, c) and round cells [51]. Interestingly, lower doses of Taxol (1 nM) induced a partial stabilisation of microtubules, more similar to that seen following the expression of the A-mutant EMTB-mCherry protein (Fig. 3a, b). This led to the stabilisation of interphase microtubules, the formation of microtubule clumps during prometaphase and to the transient formation of multipolar spindles (Fig. 3a–c) — most of which resolved prior to anaphase. These data make clear the importance of removing the population of interphase microtubules prior to assembly of the spindle.

### Step 2: dilution of the tubulin pool contributes to the loss of microtubule polymer levels during the transition from prophase to prometaphase

**Fig. 4:**
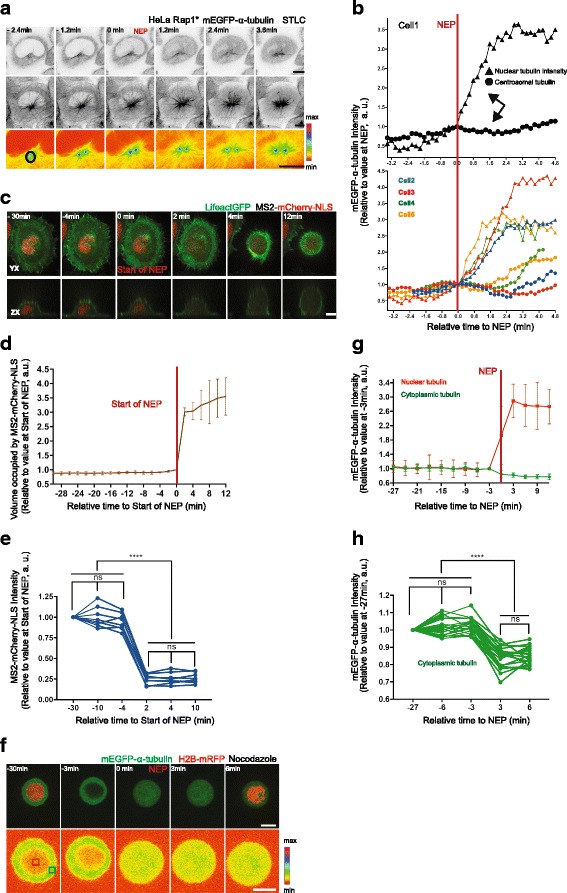
Loss of the nuclear-cytoplasmic compartment barrier is associated with the loss of tubulin polymer. **a** Representative time-lapse confocal images of a HeLa cell during mitotic entry stably expressing H2B-mRFP (not imaged) and mEGFP-α-tubulin and transiently overexpressing Rap1* treated with *S*-trityl-l-cysteine (*STLC*). *Upper panel*: upper single section (inverted greyscale) of mEGFP-α-tubulin to visualise NEP, *middle panel*: *x*-*y* maximum projection images of mEGFP-α-tubulin (inverted greyscale) to visualise microtubules, *lower panel*: sum projection of mEGFP-α-tubulin sections around the centrosome (pseudo-color, spectra LUT) to visualise centrosomal microtubules. **b** Changes in centrosomal microtubule levels (*circles*) relative to NEP (nuclear tubulin, *triangles*) for one cell (shown in **a**, *upper panel*) and for equivalent four cells from two independent experiments (*lower panel*). **c** Representative time-lapse confocal images of a HeLa cell expressing MS2-mCherry-NLS (nuclear marker) and Lifeact-GFP during mitotic entry. *Lower panel* shows lateral projections. **d** Quantification of volume occupied by MS2-mCherry-NLS signal in HeLa cells similar to that in **c** (eight cells, two independent experiments). **e** Quantification of MS2-mCherry-NLS intensity. Median of MS2-mCherry-NLS signal for cells in **d** at indicated time points. Repeated measures ANOVA with the Greenhouse-Geisser correction, Tukey's multiple comparisons test with individual variances computed for each comparison, ****P < 0.0001. **f** Representative time-lapse confocal images of a HeLa cell stably expressing H2B-mRFP (shown in the *upper panel* at –30 min and 6 min) and mEGFP-α-tubulin during mitotic entry, following treatment with Nocodazole in non-adherent chambers (*upper panel*: *x*-*y* maximum projection, *lower panel*: single section, pseudo-color, spectra LUT). **g** Quantification of median mEGFP-α-tubulin signal in cells similar to that in **f** (eight cells filmed at high resolution using spinning disc confocal microscope, one independent experiment). **h** Quantification of median mEGFP-α-tubulin signal in cells similar to that in **f** at indicated time points (20 cells filmed at low resolution with wide-field microscope, two independent experiments). Repeated measures ANOVA with the Greenhouse-Geisser correction, Tukey's multiple comparisons test, with individual variances computed for each comparison, ****P < 0.0001. For measurement details see Methods. Graphs show mean and SD. Scale bars represent 10 μm

Having established a role for Ensconsin/MAP7 in the destabilisation of interphase microtubules during prophase, we wanted to determine the cause of the sudden depolymerisation of remaining microtubules observed during loss of the nuclear-cytoplasmic compartment boundary (Fig. 1a–e, Additional file 1E). To do so, we imaged flat HeLa cells expressing histone-2B-mRFP and mEGFP-α-tubulin at a high temporal resolution (taking a frame every 0.2 min). We also pre-treated cells with *S*-trityl-l-cysteine (STLC) (to block centrosomes separation) to facilitate our ability to image both centrosomally nucleated microtubules and peripheral interphase microtubules (Fig. 4a). Under these conditions, the marked influx of mEGFP-α-tubulin into the nucleus at NEP was accompanied by the loss of residual interphase microtubules and with a transient dip in the levels of mEGFP-α-tubulin polymer at the centrosomes (Figs. 1 and 4a, b). Strikingly, however, microtubule polymer levels at the centrosomes quickly recovered in the ensuing minutes, leading to the formation of the mitotic spindle. This momentary reversal in the steady accumulation of centrosomally nucleated microtubules would be hard to explain based on regulation by mitotic kinase activity alone. We therefore considered an alternative model, whereby the sudden change in tubulin heterodimer concentration induced by permeabilisation of the nuclear envelope causes a relatively rapid change in the kinetics of tubulin polymer assembly — since the concentration of tubulin heterodimer is a key factor in determining the dynamic behaviour of microtubules [53, 54]. In vitro, a decrease in tubulin heterodimer concentration leads to an increase in the frequency of catastrophe events [53] and to a decrease in the rate of microtubule nucleation [54]. To determine whether the changes in tubulin heterodimer concentration that occur during NEP are of the right order to explain the observed changes in microtubule assembly, we used a nuclear-localised protein, MS2-mCherry-NLS, to measure the volume of the nucleus relative to that of the entire cell during the transition into mitosis [55]. On average, NEP led to a threefold (±0.16) increase in the volume occupied by the MS2-mCherry-NLS signal (Fig. 4c, d) and a similar fourfold (±1.6) decrease in the MS2-mCherry-NLS concentration (Fig. 4e). To measure the extent of tubulin heterodimer dilution, we also performed the converse analysis in Nocodazole-treated HeLa cells expressing mEGFP-labelled tubulin and histone-2B-mRFP mEGFP-α-tubulin. To facilitate the accurate measurement of nuclear and cell volume under these conditions, we imaged rounded cells held in non-adhesive chambers (Fig. 4f). In these cells, NEP was associated with a decrease in the peripheral mEGFP-α-tubulin signal by an average of 18 ± 6% (Fig. 4f–h). Taken together, these data confirm that loss of the nuclear-cytoplasmic compartment boundary is accompanied by a significant reduction in the concentration of tubulin heterodimers.

**Fig. 5:**
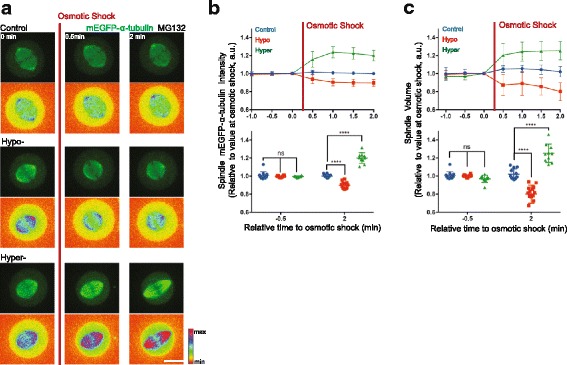
Changes in volume are sufficient to induce changes in tubulin polymer levels. **a** Representative time-lapse confocal images (*x*-*y* maximum projection, *lower panel*: pseudo-color, spectra LUT) of HeLa cells stably expressing H2B-mRFP (not imaged) and mEGFP-α-tubulin showing changes in spindle mEGFP-α-tubulin intensity and spindle volume upon hypo- or hyper-osmotic shock relative to a control. Scale bar represents 10 μm. **b** Quantification of changes in spindle mEGFP-α-tubulin intensity induced by osmotic shock. Mean intensity of spindle mEGFP-α-tubulin intensity was measured by rendering mitotic spindles in three dimensions (3D) using Imaris software before and after control (*blue*, 12 cells), hypo (*red*, 14 cells) or hyper (*green*, 10 cells) osmotic shock treatment (two independent experiments). *Lower panel*: comparison between –0.5 min and 2 min relative to osmotic shock treatment. Repeated measures two-way ANOVA, Dunnett's multiple comparisons test, ****P = 0.0001. **c** Quantification of changes in spindle volume induced by osmotic shock. Spindle volume was measured by rendering mEGFP-α-tubulin signal in 3D in Imaris before and after control (*blue*, 12 cells), hypo (*red*, 14) or hyper (*green*, 10 cells) osmotic shock treatment (two independent experiments). *Lower panel*: comparison between –0.5 min and 2 min relative to osmotic shock treatment. Repeated measures two-way ANOVA, Dunnett's multiple comparisons test, ****P = 0.0001

To determine, empirically, how a relatively sudden 15–20% dilution of the tubulin pool is likely to affect microtubule polymer levels in mitosis, we established an assay enabling us to induce a similar, rapid reduction in the in vivo concentration of tubulin heterodimers using hypo-osmotic shock (Additional file 4A). While the control treatment did not trigger any significant changes in either mEGFP-α-tubulin intensity or in the cell diameter, hypo-osmotic shock induced changes in tubulin heterodimer concentration that were quantitatively similar to those observed at NEP: a 6.3 ± 2.5% increase in the cell diameter and a concomitant 16.2 ± 2.8% decrease in the mEGFP-α-tubulin signal within 2 min of the shock (Additional file 4A–C). To test whether this is sufficient to reduce microtubule polymer levels as expected under this model, we repeated this analysis in cells that had been arrested in mitosis using MG132 treatment. Under these conditions, a dilution of tubulin dimers equivalent to that observed at NEP triggered a dramatic reduction in the levels of tubulin polymer. Within 2 min of the shock, we observed an 11.4 ± 3.7% decrease in the mEGFP-α-tubulin signal within the mitotic spindle and an accompanying 20 ± 10% decrease in the mitotic spindle volume (Fig. 5a–c). Note that hypo-osmotic shock had little visible affect on microtubules in interphase cells (Additional file 4D).

To carry out the converse experiment, we established a protocol to increase the tubulin dimer concentration using hyper-osmotic shock (Additional file 4A–C, see Methods). This increased the cytoplasmic mEGFP-α-tubulin signal on average by 53.7 ± 3.6% (Additional file 4A–C) and resulted in an increase in the spindle mEGFP-α-tubulin signal by an average of 20.0 ± 5.9%, together with a 25.2 ± 10.2% increase in the mitotic spindle volume within 2 min of the shock (Fig. 5a–c). As these data make clear, the loss of the nuclear-cytoplasmic compartment boundary induces rapid changes in the concentration of cytoplasm that are likely to have a significant impact on a wide range of cellular processes, including microtubule disassembly. Moreover, this will be compounded by the osmotic swelling associated with NEP, which is reported to be of the order of 15–20% [56, 57].

Finally, having already shown that it is important to remove Ensconsin/MAP7 from microtubules before NEP for proper spindle assembly, we wanted to test the effects of driving premature NEP in cells prior to the full inactivation of Ensconsin/MAP7 (Additional file 4E, F). To do so, we triggered nuclear envelope rupture via laser ablation in prophase cells that had been depleted of Lamin A and the ESCRT-III protein CHMP3 using RNAi to prevent the rapid resealing of the nuclear envelope. In cells that were close to NEP (identified by bright centrosomes, Additional file 4E, F, late prophase), the premature rupture of nuclear envelope induced fast depolymerisation of microtubules and a decrease in α-tubulin-GFP signal on centrosomes — as was seen in control cells (Additional file 4E, F, Control). By contrast, in cells with dimmer centrosomes (likely those with lower levels of Cdk1/cyclin B and higher levels of Ensconsin/MAP7 associated with microtubules, Additional file 4E, F, early prophase), we could not observe significant levels of microtubule depolymerisation upon premature rupture of the nuclear envelope despite the rise in the nuclear pool of tubulin monomer. Thus, the removal of Ensconsin/MAP7 (and potentially other factors) from microtubules before NEP likely aids the fast depolymerisation of interphase microtubules triggered by NEP to ensure faithful spindle morphogenesis. This makes clear the importance of microtubule disassembly being carried out as a two-step process.

## Discussion

### The dynamics of interphase microtubule disassembly

In this study, we explore how interphase microtubule disassembly is influenced by mitotic entry. While this is a central part of the spindle assembly process, the molecular mechanisms involved remain poorly understood because of the difficulties of imaging microtubules in cells undergoing mitotic rounding. For this reason, the remodelling of the microtubule cytoskeleton at mitotic entry has been studied in most detail in Ptk1 and LLCPk1 cells [6, 58], which have few chromosomes and remain relatively flat during mitosis, rather than in human cells. Here, in order to visualise the dynamics of microtubule remodelling in live human cells, we used Rap1* overexpression to flatten HeLa cells carrying fluorescently tagged proteins. In this way, we were able to analyse the dynamics of microtubule remodelling upon entry into mitosis in detail to reveal the following:

1. The activation of centrosomal nucleation is accompanied by the destabilisation and gradual loss of interphase microtubules.
2. The activation of centrosomal nucleation is not mechanistically coupled to the disassembly of interphase microtubules.
3. The loss of the nuclear-cytoplasmic compartment boundary is associated with dilution of the tubulin heterodimer pool, with the sudden loss of residual interphase microtubules [6, 58], and with a transient dip in the levels of centrosomally nucleated microtubules.

### The mechanism contributing to interphase microtubule disassembly

Our analysis points to there being two distinct processes at play. First, the rise of Cdk1/cyclin B activity that drives entry into mitosis induces the phosphorylation of a microtubule-stabilising protein, Ensconsin/MAP7. This reduces its affinity for microtubules [39, 43], making interphase microtubules susceptible to subsequent disassembly. Although these data suggest a specific role for Cdk1/cyclin B in this regulation, the experiments performed do not exclude a role for other mitotic kinases (including Nek2). In addition, even though our data point to the importance of the timely removal of Ensconsin/MAP7 from interphase microtubules, it is likely that the mitotic phosphorylation of other microtubule-binding proteins, which can change microtubule stability, also contributes to this process. Indeed, previous work has suggested that the phosphorylation of MAP4 by Cdk1 reduces its ability to stabilise microtubules [59, 60]. In addition, Cdk1 has been shown to be a regulator of plus-end tracking proteins (+TIPs) such as EB2 and CLASP2, which play an important role in regulation of microtubule dynamics and mitotic progression [61, 62]. Moreover, Plk1, another major mitotic kinase, has been shown to stimulate microtubule polymerisation activity of MCAK, a key regulator of microtubule dynamics [63, 64] and of the cytoplasmic linker protein (CLIP)-170, which is involved in timely formation of kinetochore-microtubule attachments [65]. Moreover, it has been shown that SLAIN2 and STIM1 (+TIPs) are phosphorylated and dissociated from microtubule plus ends during mitosis [66, 67]. Even the minus-end-targeting proteins (–TIPs), such as CAMSAP2, were found to be removed from the microtubules in prophase due to extensive phosphorylation [66, 68]. Another minus-end-targeting protein, CAMSAP3, also has been reported to be associated with microtubules predominantly in interphase [43]. Thus, the activation of Cdk1/cyclin B is likely to trigger the remodelling of the microtubule cytoskeleton through several parallel processes. Although this is the case, our data show that while the rise in mitotic kinase activity sets the stage for the depolymerisation of microtubules following the loss of the nuclear-cytoplasmic compartment boundary, it is not in itself sufficient for the complete disassembly of interphase microtubules.

As our analysis makes clear, the profound changes in cytoskeletal organisation accompany loss of the nuclear-cytoplasmic compartment barrier — the event previously suggested to commit cells to mitosis [27]. NEP triggers a relatively rapid but transient loss of microtubule polymer from the centrosomes, together with loss of the residual interphase microtubules. The transient loss of centrosomal microtubules is particularly surprising here, since this is the population of microtubules required to form the nascent spindle (Fig. 1). In looking for alternative mechanisms to explain this behaviour, we focused on the role of loss of the nuclear compartment barrier itself, since this is expected to be accompanied by a relatively sudden reduction in the concentration of tubulin heterodimers [19, 53, 54] (which we measured as ~ 15–20%). In line with this idea, osmotic shocks designed to mirror or reverse the observed changes in tubulin concentration that accompany NEP were found to have a profound impact on microtubule polymer levels in mitotic cells. In general, the extent of the dilution in tubulin heterodimer (and other exclusively cytoplasmic proteins) accompanying mitotic entry will depend on two factors: (1) the relative size of the nuclear and cytoplasmic compartments, which varies across mammalian cell types (being very large in embryonic stem cells), and (2) the extent of mitotic cell swelling, which accompanies loss of the nuclear-cytoplasmic compartment barrier [56, 57]. When combined, our data suggest that these two factors will lead to a profound and functionally significant change in tubulin concentration. Thus, NEP will act in concert with alterations in the activity of microtubule-associated proteins that are triggered by mitotic entry to change microtubule dynamics [18], especially since many of these proteins are preferentially sequestered within a single compartment (the cytoplasm or nucleus) in interphase.

### Importance of interphase microtubule disassembly

In the course of our analysis, we also tested the functional importance of this being a two-step process of microtubule destabilisation. We did this by exploring the consequences of blocking the ability of mitotic kinases to induce the release of Ensconsin/MAP7 from microtubules in prophase. In line with this being an important event, the expression of a non-phosphorylatable form of the Ensconsin/MAP7 microtubule-binding domain interfered with the normal process of interphase microtubule disassembly. Strikingly, the microtubules that remained formed clusters (perhaps as a result of dynein/dynactin-dependent movement [58]), which resembled those induced by the microtubule-stabilising compound Taxol, a drug widely used in cancer treatment [49]. These clusters were not incorporated into the nascent spindle. In fact, under some conditions, they perturbed spindle assembly, leading to the formation of multipolar spindles and divisions — like those seen in response to clinically relevant concentrations of Taxol [69, 70]. Interestingly, in this light, these data also provide an explanation for previously published observations that Taxol only induces multipolar spindle formation when added before cells enter mitosis or during early prophase [49–51] — not when added after microtubule cytoskeleton remodelling [71, 72]. Thus, the destabilisation of microtubules prior to NEP is a critical part of the process of interphase microtubule disassembly and is a necessary prelude to spindle morphogenesis. Interestingly, similar clumps were observed in cells in which premature NEP was induced via laser ablation prior to interphase microtubule disassembly.

This two-step regulation of microtubule dynamics at mitotic entry has an additional consequence. As interphase microtubules are gradually lost during the course of prophase, the tubulin that is released is incorporated into microtubules nucleated during the process of centrosome maturation. These grow to long lengths, preserving the overall levels of tubulin polymer (Fig. 1, [6]). While the consequences of this are not completely clear, increases in the lengths of centrosomally nucleated microtubules may aid the separation and positioning of centrosomes, since cells at this stage have yet to complete mitotic rounding [73]. Then, as a consequence of NEP, the population of long centrosomally nucleated microtubules is quickly replaced by short, dynamic centrosomal microtubules that are used to search out and capture kinetochores. At the same time, short astral microtubules function to refine spindle positioning in the confines of the rounded metaphase cell. Thus, while it remains to be tested, it is possible that this type of two-step regulation enables centrosomal microtubules to perform distinct functions during prophase and prometaphase. The timing of nuclear envelope permeabilisation may therefore be important in mediating this transition between modes of microtubule remodelling.

## Conclusions

In summary, our analysis reveals a two-step process by which interphase microtubules are disassembled upon entry into mitosis. The disassembly of interphase microtubules frees up tubulin heterodimers for its incorporation into the spindle and aids mitosis by preventing non-centrosomal microtubules from interfering with spindle morphogenesis. This need to disassemble interphase microtubules in prophase may, in part, explain the toxicity of Taxol in dividing cancer cells [70]. Further, this study shows that the changes in cell organisation that accompany mitosis are driven both by changes in the regulation of individual proteins by phosphorylation and by the profound changes in the make-up of the cytoplasm that accompany the transition into mitosis [74].

## Methods

### Cell culture, RNAi, DNA transfection, mutagenesis, immunoblotting and drug treatments

Unlabelled HeLa Kyoto cells and HeLa stable cell lines stably expressing histone-2B-RFP and mEGFP-α-tubulin [33] and GFP-α-tubulin [46] were cultured at 37 °C in a humidified incubator under 5% CO_2_ in Dulbecco’s modified Eagle’s medium (DMEM, Gibco) with 10% fetal bovine serum (Gibco) and 1% Pen-Strep (Sigma-Aldrich). Unlabelled MCF10A cells were cultured at 37 °C in a humidified incubator under 5% CO_2_ in DMEM: Nutrient Mixture F-12 (DMEM/F12, Gibco) with 5% horse serum and 1% Pen-Strep (Sigma-Aldrich), and with the following supplements: 20 ng/ml human Epithelial Growth Factor (hEGF, Roche), 0.5 mg/ml hydrocortisone (Sigma), 100 ng/ml cholera toxin (Sigma) and 10 μg/ml insulin (Sigma). The cells were tested and are mycoplasma-free.

Lipofectamine 2000 (Invitrogen) was used for siRNA transfections according to the manufacturer's protocol. Cells were analysed 48 h after transfection. siRNAs were used to knock down Ensconsin/MAP7 (CUACAAAGCUGCACACUCU, UCAGAGAAACGGUGAUAUA, CCAUGAAUCUUUCGAAAUA, all Dharmacon), Cep192 [38] (AGC AGC UAU UGU UUA UGU UGA AAA U (Eurofins custom designed, transfected cells were identified by abolished mEGFP-α-tubulin signal at centrosomes), Lamin A (CAGGCAGTCTGCTGAGAGGAA, Qiagen) and CHMP3 [75] (AAA GCA UGG ACG AUC AGG AAG), and compared to a non-targeting control (AllStars Negative Control siRNA, Qiagen).

HeLa cells were transfected with pRK5-Rap1[Q63E] (Rap1*, cells transfected with Rap1* were identified by their failure to round up at the mitotic entry) [34], EMTB-mCherry (a kind gift from J. Pines’ lab), A/E-EMTB-mCherry (synthesised by Eurofins), where the following amino acids were mutated to alanine/glutamic acid compared to wt-EMTB: Cdk1 sites: S169, S209, S219, T231, S254, T277; Nek2 sites: S165, S188, S202, S240 (positions refer to Ensconsin/MAP7 canonical sequence, UniProt identifier: Q14244-1), MS2-mCherry-NLS [55] together with Lifeact-GFP (a kind gift from Ewa Paluch’s lab) or EMTB-3XGFP (a gift from William Bement, Addgene plasmid # 26741) using Fugene HD (Promega) according to the manufacturer’s instructions. Cells were analysed 24 h after transfection.

Depletion of Ensconsin/MAP7 was verified by immunoblotting. siRNA-treated cells were lysed with 1XSB (Invitrogen) and loaded onto an SDS-PAGE gel before transfer onto an Immobilon-P (Millipore) membrane by wet western blotting. Membranes were blocked in 5% bovine serum albumin (BSA) in tris-buffered saline and Tween 20 (TBST) for 1 h, incubated overnight at 4 °C with primary antibodies, and for 1 h at room temperature with secondary antibodies. Antibodies were used at the following dilutions: Ensconsin/MAP7 1:2500 (Proteintech, 13446-1-AP), α-tubulin 1:5000 (DM1A, Sigma) and HRP-conjugated secondary antibodies 1:5000 (Dako). Results were visualised using an ImageQuant LAS4000 system.

Drugs were used at the following concentrations: 100 ng/ml Nocodazole (Sigma), 1 or 2 nM Taxol (Sigma), 1 μM MG132 (Sigma), 10 μM STLC (Sigma), 9 μM RO-3306. Cells were incubated at least 1 h with the drugs (except RO-3306, which was left on cells only for 15 min) before live cell imaging.

### Live cell imaging

For confocal live cell imaging, cells were seeded on glass-bottomed dishes (MatTek) coated with 10 mg/ml fibronectin (Sigma) and imaged in Leibovitz's L-15 Medium (Gibco) using an UltraView Vox (Perkin Elmer) spinning disc confocal microscope with a 60X (NA 1.4) or 100X (NA 1.4) oil objective equipped with a temperature-controlling environmental chamber. Images were acquired using a Hamamatsu ImagEM camera and Volocity software (Perkin Elmer). Wide-field live cell imaging was done using a Zeiss Axiovert 200 M microscope with a 20X objective (NA 0.4) equipped with a temperature-controlling environmental chamber, and images acquired using a Hamamatsu Orca AG camera and Volocity software (Perkin Elmer).

Nuclear envelope rupture experiments were done using an UltraView Photokinesis Device with 405 nm laser.

For the tubulin dilution calculations at NEP, cells were seeded in polydimethylsiloxane (PDMS) chambers incubated overnight with 0.1 mg/ml polyethylene glycol (PEG, Sigma) dissolved in 10 mM 4-(2-hydroxyethyl)-1-piperazineethanesulfonic acid (HEPES), pH 7.4.

Osmotic shock was induced either by diluting the imaging media by 50% with deionised water (hypo-osmotic shock) or by adding 4 mM sorbitol (Sigma) solution to an end concentration of 1.3 mM (hyper-osmotic shock). For the control experiments, imaging medium was added.

### Immunofluorescence

For immunostaining, cells were plated on fibronectin-coated glass chambers and either fixed with 4% formaldehyde for 20 min and then permeabilised with 0.2% Triton-X in phosphate-buffered saline (PBS) for 5 min, or fixed and permeabilised at the same time with PEMT buffer (0.1 M piperazine-*N*,*N*’-bis(2-ethanesulfonic acid) (PIPES), 1 mM MgCl_2_, 1 mM ethylene glycol tetraacetic acid (EGTA), 0.2% Triton-X, 4% paraformaldehyde (PFA)), then blocked with 5% BSA in PBS for 30 min and treated with primary and secondary antibodies for 1 h at room temperature. Primary antibodies were used at the following dilutions: tubulin 1:1000 (DM1A, Sigma-Aldrich), Ensconsin/MAP7 (MAP7, Proteintech, 13446-1-AP) 1:100-200, mCherry 1:500 (Abcam). Secondary anti-rabbit IgG and anti-mouse IgG antibodies (Molecular Probes) tagged with Alexa-Fluor 488 or 546 were used at 1:500 and 4’,6-diamidino-2-phenylindole (DAPI, Invitrogen) at 1:2000. Immunostained cells were mounted with FluorSave (Calbiochem) and imaged on a Leica SPE confocal microscope with a 63X lens (NA 1.3) or on an UltraView Vox (Perkin Elmer) spinning disc confocal microscope with 60X (NA 1.4).

### Image processing and analysis

Displayed images were processed using ImageJ where necessary, and the brightness was changed uniformly across the field.

Changes in centrosomal microtubule levels relative to NEP were quantified in the following way. The median intensity of the mEGFP-α-tubulin signal in a 15 × 15 pixel oval around the centrosomes and the background signal in a 3 × 3 pixel box close to the centrosomes were manually measured in the sum projection images around one centrosome per cell, which did not move much in the *z*-direction with ImageJ, as shown in Fig. 1d. As shown in Fig. 4b, the median intensity of the mEGFP-α-tubulin signal in a 20 × 20 or 30 × 30 pixel oval around both centrosomes was measured if the centrosomes stayed close enough to each other during the whole course of the movie. Otherwise, a 19 × 19 pixel oval around one centrosome was used for the measurements. For other data, a custom MATLAB code was used to measure the mEGFP-α-tubulin signal at the centrosomes semi-automatically. Centrosomes and points for background measurements close to the centrosomes were chosen manually, and the code calculated the mean of mEGFP-α-tubulin in the cylinder with 15 pixels (for centrosomes) and 5 pixels (for background) diameter and 3 section height. The values displayed in the graphs are background subtracted and normalised by the values at NEP or at –30 min before NEP, as indicated.

Changes in non-centrosomal microtubule levels relative to NEP were quantified as follows. The median/variance of the mEGFP-α-tubulin signal in a 30 × 30 pixel box at the periphery of a cell and outside a cell (background) was manually measured with ImageJ (see Fig. 1 and Additional file 1A). The values displayed in the graphs are background subtracted and normalised by the values at NEP or at –30 min before NEP, as indicated. In addition, the cytoplasmic mEGFP-α-tubulin intensity (value at 6 min after the NEP) was subtracted before normalisation (except in the graphs in Fig. 1 and Additional file 1). In Figs. 1d and 2b the median of mEGFP-α-tubulin was measured only at one location per cell in sum projection images of basal sections. Otherwise, the median/variance of mEGFP-α-tubulin was measured at two locations per cell in the single sections.

For nuclear tubulin, the median intensity of the mEGFP-α-tubulin signal was measured in the box (size indicated in figure legends) in the nucleus and outside the cell in the single sections. The values displayed in the graphs are background subtracted and normalised as indicated in the figures.

To measure tubulin dilution in the cells treated with Nocodazole in non-adherent chambers, the median intensity of mEGFP-α-tubulin was measured in a 6 × 6 pixel box at two locations at the periphery of cells and outside the cells (background) in single sections in high resolution movies (60X, spinning disc). In low resolution movies (20X, wide-field microscope) the median mEGFP-α-tubulin signal was measured in a 4 × 4 pixel box at four locations at the periphery of the cells and at one location outside the cells (background). The values displayed in the graphs are background subtracted and normalised as indicated in the figures.

To measure changes in the MS2-mCherry-NLS concentration, the median intensity of the MS2-mCherry-NLS signal was measured in a 20 × 20 pixel box at two locations in the nucleus and outside the cell (background) in the single sections. The values displayed in the graphs are background subtracted and normalised as indicated in the figure.

The volume occupied by MS2-mCherry-NLS was measured using a custom MATLAB code, developed to be independent of the overall mCherry intensity. Briefly, a point within the volume occupied by MS2-mCherry-NLS was found using a difference-of-Gaussians (DoG) filter. This was then expanded to find the boundary of the volume using a gradient watershed algorithm in three dimensions (3D). Cells were tracked from frame to frame using the Munkres algorithm; the minimal movement of cells between frames allowed trivial robust tracking.

To measure tubulin, the cytoplasmic mEGFP-α-tubulin intensity upon control, hypo- and hyper-osmotic shock treatments was quantified in the following way in 3D: a 30-pixel-width line was drawn across the cell in *xy* and, using the FIJI “*KymoResliceWide*” plugin, average intensity values across the width were calculated for each section, creating kymographs in the z-direction for every time point. Then a maximum size box according to the cell size was drawn on the kymographs and mean intensities were calculated for every time point.

The maximum diameter of the cells upon control, hypo- and hyper-osmotic shock treatments was calculated by manually identifying contours of the cells at every time point in maximum projection images and measuring the corresponding Feret diameter. The spindle mEGFP-α-tubulin intensity and spindle volume were calculated via rendering of the spindle mEGFP-α-tubulin signal in 3D using Imaris (Bitplane).

Graphs were produced and statistical analysis (as indicated in the figure legends) performed using GraphPad Prism.

In the overlay images in Fig. 2, the signal intensities were adjusted to remove the cytoplasmic background signal.

## Additional files

Additional file 1: Disassembly of interphase microtubules begins prior to NEP and accelerates at NEP. A) Representative confocal image (*x*-*y* maximum projection) of an H2B-mRFP mEGFP-α-tubulin HeLa cell during mitotic progression. B) Representative confocal image (*x*-*y* maximum projection) of H2B-mRFP (not shown) mEGFP-α-tubulin HeLa cells (inverted greyscale) transiently overexpressing Rap1* entering mitosis (the same cell as in Fig. 1) and a control cell that remains in interphase during the course of the movie. Boxed areas show regions that were used for quantifications in C–E. Scale bar represents 10 μm. C) Changes in non-centrosomal microtubule levels in the cells entering mitosis (*blue*, the same as in Fig. 1d) vs. interphase cells (*black*, five cells from two independent experiments). Median intensity of mEGFP-α-tubulin signal was measured as in Fig. 1d. Graph shows mean and SD. Changes in non-centrosomal microtubule levels in the cells entering mitosis (*blue*, the same as in Fig. 1e) vs. interphase cells (*black*, 13 cells from four independent experiments). Median (D) and variance (E) of mEGFP-α-tubulin signal were measured as in Fig. 1e. F) Changes in centrosomal microtubule levels. Mean of mEGFP-α-tubulin signal at the centrosomes was measured as in Fig. 2b in the cells entering mitosis at –30, –20, –2 min before NEP and 2, 4 min after NEP (13 cells, four independent experiments, the same cells as in Fig. 1). Repeated measures ANOVA with the Greenhouse-Geisser correction, Tukey's multiple comparisons test with individual variances computed for each comparison. Shown *P = 0.039. Scale bars represent 10 μm. (PDF 1360 kb)

Additional file 2: Binding of Ensconsin/MAP7 to microtubules at mitotic entry is regulated by phosphorylation. A) Changes in non-centrosomal microtubule levels relative to NEP. Variance of α-tubulin-GFP signal measured as described in Additional file 1E for control siRNA (*blue*, five cells) and Cep192 siRNA cells (*brown*, five cells) for two experiments as in Fig. 2a–c. B) Western blot showing Ensconsin/MAP7 knockdown induced using three different siRNAs targeting Ensconsin/MAP7. C) Representative confocal images (*x*-*y* maximum projection) of fixed HeLa cells treated with control siRNA and three siRNAs targeting Ensconsin/MAP7. Boxed areas show regions zoomed in overlays (>10 cells per condition, one experiment). D) Representative confocal images (*x*-*y* maximum projection) of fixed MCF10A cells stained to show that Ensconsin/MAP7 is removed from microtubules in prophase compared to interphase. Ensconsin/MAP7 in *red*, α-tubulin in *green* and DAPI in *blue*. Boxed areas show regions zoomed in overlays, in which intensities were adjusted to remove cytoplasmic background signal (six prophase cells, > 20 interphase cells, one experiment). E) Representative confocal images of fixed HeLa cells overexpressing Rap1* in prophase stained to show that the Wt-EMTB-mCherry as well as a corresponding phospho-mimetic mutant (E-EMTB-mCherry) are largely cytoplasmic in prophase, whereas the non-phosphorylatable form (A-EMTB-mCherry) localises to the microtubules. α-Tubulin in *green*, Wt-EMTB-mCherry, A-EMTB-mCherry, E-EMTB-mCherry in *red*. Boxed areas are zoomed, shown in inverted greyscale or in overlays, where signal intensities were adjusted to remove cytoplasmic background signal (three cells per condition, one experiment). F) Representative time-lapse confocal images (*x*-*y* maximum projection) of flat (Rap1*) HeLa cells stably expressing GFP-α-tubulin and Wt-EMTB-mCherry (*upper panel*) or its non-phosphorylatable mutant (A-EMTB-mCherry, *lower panel*) to show that the non-phosphorylatable mutant stays associated with microtubules during prophase, leading to delay in microtubule disassembly at mitotic entry even if its expression level is lower compared to Wt-EMTB-mCherry. Boxed areas show regions zoomed in inverted greyscale or in overlays, where signal intensities were adjusted to remove cytoplasmic background signal. *Black arrows* indicate interphase microtubules just before or after NEP. Scale bars represent 10 μm. (PDF 5920 kb)

Additional file 3: Failure in removal of Ensconsin/MAP7 from microtubules in prophase delays interphase microtubule disassembly and leads to an abnormal-looking mitotic spindle. Movie shows Flat (Rap1*) HeLa cell stably expressing GFP-α-tubulin (*green*) and non-phosphorylatable Ensconsin/MAP7 microtubule-binding domain (A-EMTB-mCherry, *red*) during mitotic progression. Scale bar represents 10 μm. (AVI 1091 kb)

Additional file 4: Hypo-osmotic shock can be used to mimic changes in tubulin concentration induced by NEP. A) Representative time-lapse confocal images (*x*-*y* maximum projection, *lower panel*: pseudo-color, spectra LUT) of HeLa cells stably expressing H2B-mRFP (not imaged) and mEGFP-α-tubulin, treated with Nocodazole, to show changes in cell diameter and in mEGFP-α-tubulin intensity before and after hypo- or hyper-osmotic shock treatment relative to control treatment. Quantifications of changes in mEGFP-α-tubulin intensity (B) and in cell diameter (C) induced by osmotic shock relative to control treatment. Mean intensity of mEGFP-α-tubulin signal and cell diameter was measured in cells before and after control (*blue*, seven cells), hypo- (*red*, eight cells) or hyper- (*green*, eight cells) osmotic shock treatments (two independent experiments) as described in Methods. *Lower panels* show comparison between values at –0.5 min and 2 min relative to osmotic shock treatments. Repeated measures two-way ANOVA, Dunnett's multiple comparisons test, ****P = 0.0001. D) Representative time-lapse confocal images (*x*-*y* maximum projection, *lower panel*: pseudo-color, spectra LUT) of HeLa cells stably expressing H2B-mRFP (not imaged) and mEGFP-α-tubulin showing that hypo-osmotic shock affects mitotic spindle, whereas it does not have impact on interphase microtubules. *White arrows* indicate mitotic spindle. E) Representative time-lapse confocal images (*x*-*y* maximum projection) of HeLa cells stably expressing H2B-mRFP and mEGFP-α-tubulin and transiently overexpressing Rap1 treated with Lamin A siRNA and ESCRT-III siRNA during mitotic entry. Boxed areas are zoomed below. Control cell represents a Lamin A siRNA and ESCRT-III siRNA treated cell entering mitosis. The following cells represent accordingly a cell where nuclear envelope rupture was induced in late prophase (close to NEP) followed by immediate disassembly of microtubules and a cell where nuclear envelope rupture was induced in early prophase without triggering immediate disassembly of microtubules. F) Quantifications of timing of changes in centrosomal and non-centrosomal microtubule levels relative to NEP or to nuclear envelope (NE) ablation in cells represented in E as described in Fig. 2b. Scale bars represent 10 μm. (PDF 6682 kb)

## Abbreviations

MAP: microtubule-associated protein
NEP: nuclear envelope permeabilisation
