# Supplementary figures and images for: Two-step interphase microtubule disassembly aids spindle morphogenesis
Source: BMC Biol. 2018 Jan 23;16:14. doi: 10.1186/s12915-017-0478-z (PMC5778756; doi:10.1186/s12915-017-0478-z)

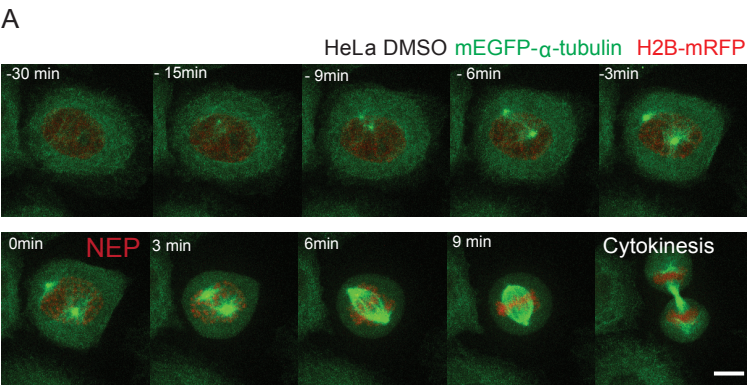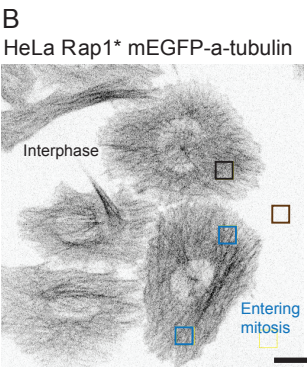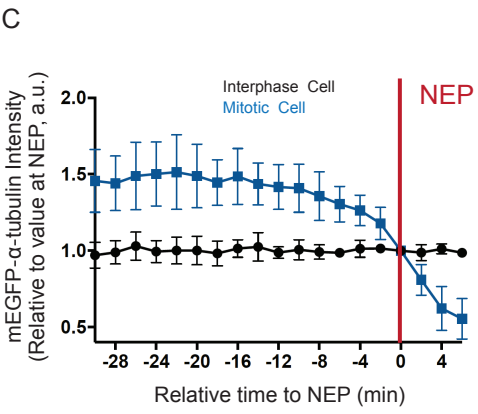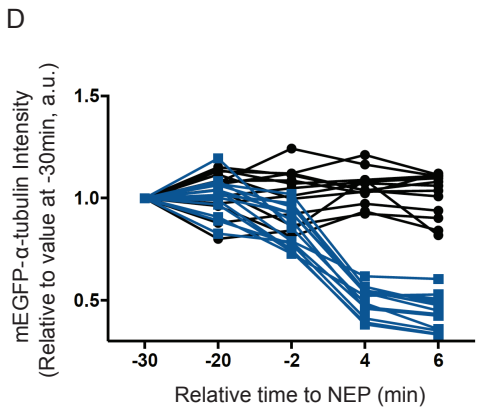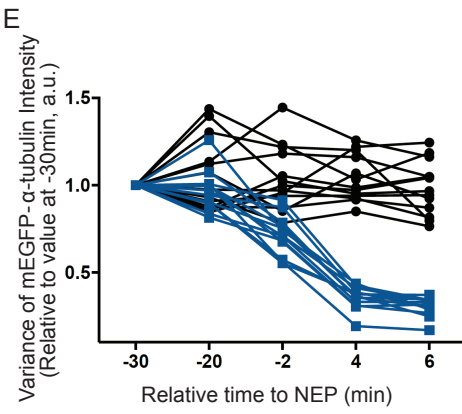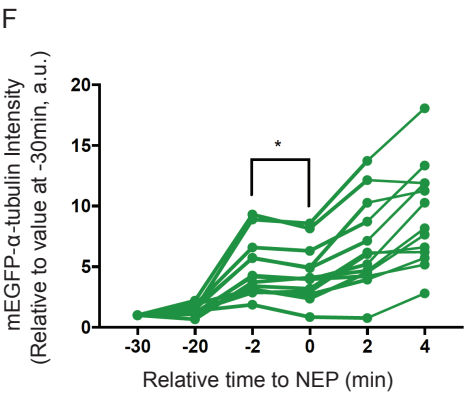

Supplement: Supplementary file 1 — Disassembly of interphase microtubules begins prior to NEP and accelerates at NEP. A) Representative confocal image (x-y maximum projection) of an H2B-mRFP mEGFP-α-tubulin HeLa cell during mitotic progression. B) Representative confocal image (x-y maximum projection) of H2B-mRFP (not shown) mEGFP-α-tubulin HeLa cells (inverted greyscale) transiently overexpressing Rap1* entering mitosis (the same cell as in Fig. 1) and a control cell that remains in interphase during the course of the movie. Boxed areas show regions that were used for quantifications in C–E. Scale bar represents 10 μm. C) Changes in non-centrosomal microtubule levels in the cells entering mitosis (blue, the same as in Fig. 1d) vs. interphase cells (black, five cells from two independent experiments). Median intensity of mEGFP-α-tubulin signal was measured as in Fig. 1d. Graph shows mean and SD. Changes in non-centrosomal microtubule levels in the cells entering mitosis (blue, the same as in Fig. 1e) vs. interphase cells (black, 13 cells from four independent experiments). Median (D) and variance (E) of mEGFP-α-tubulin signal were measured as in Fig. 1e. F) Changes in centrosomal microtubule levels. Mean of mEGFP-α-tubulin signal at the centrosomes was measured as in Fig. 2b in the cells entering mitosis at –30, –20, –2 min before NEP and 2, 4 min after NEP (13 cells, four independent experiments, the same cells as in Fig. 1). Repeated measures ANOVA with the Greenhouse-Geisser correction, Tukey's multiple comparisons test with individual variances computed for each comparison. Shown *P = 0.039. Scale bars represent 10 μm. (PDF 1360 kb) [file 12915_2017_478_MOESM1_ESM.pdf]

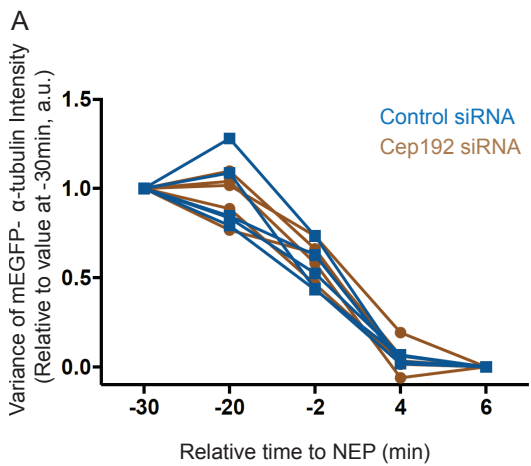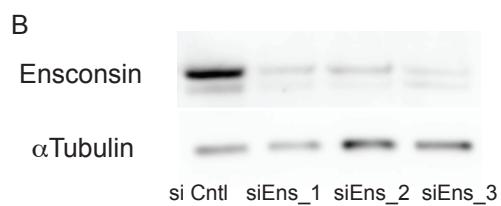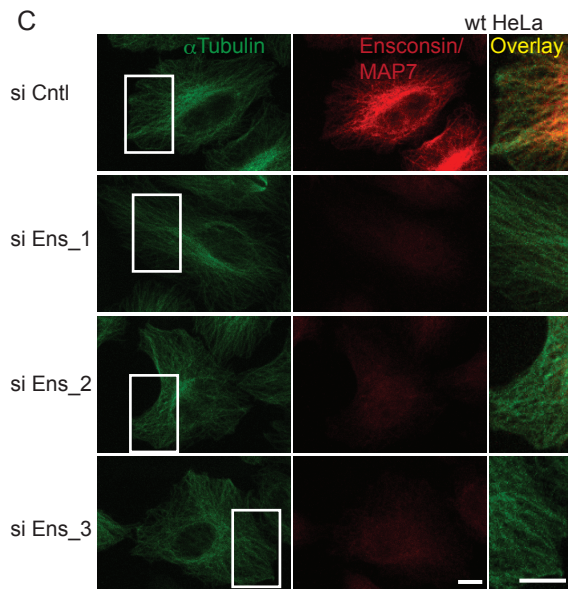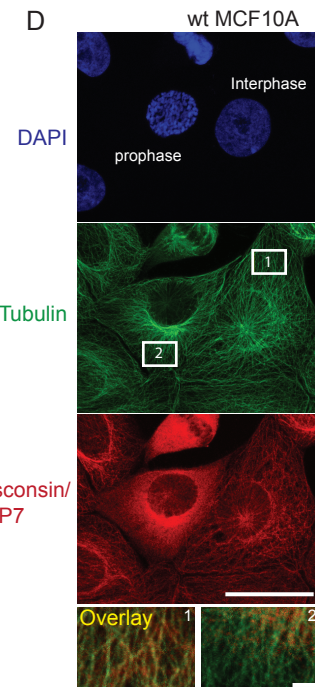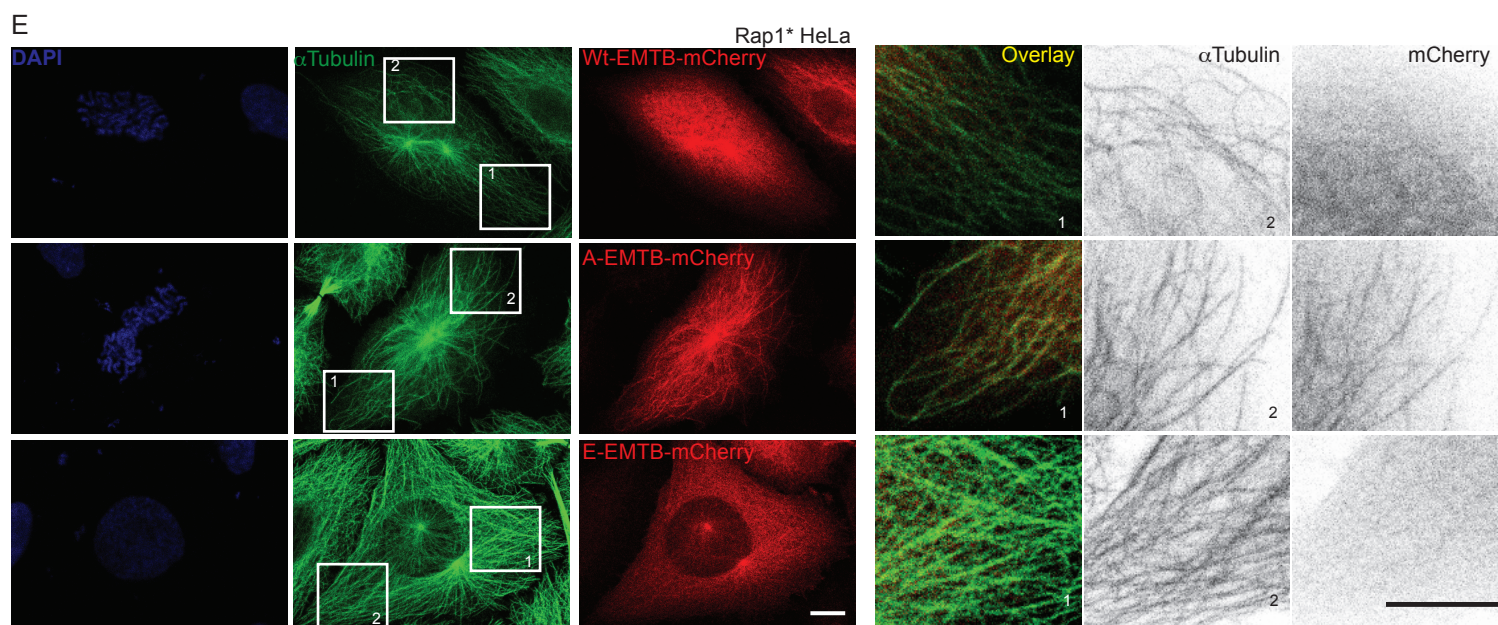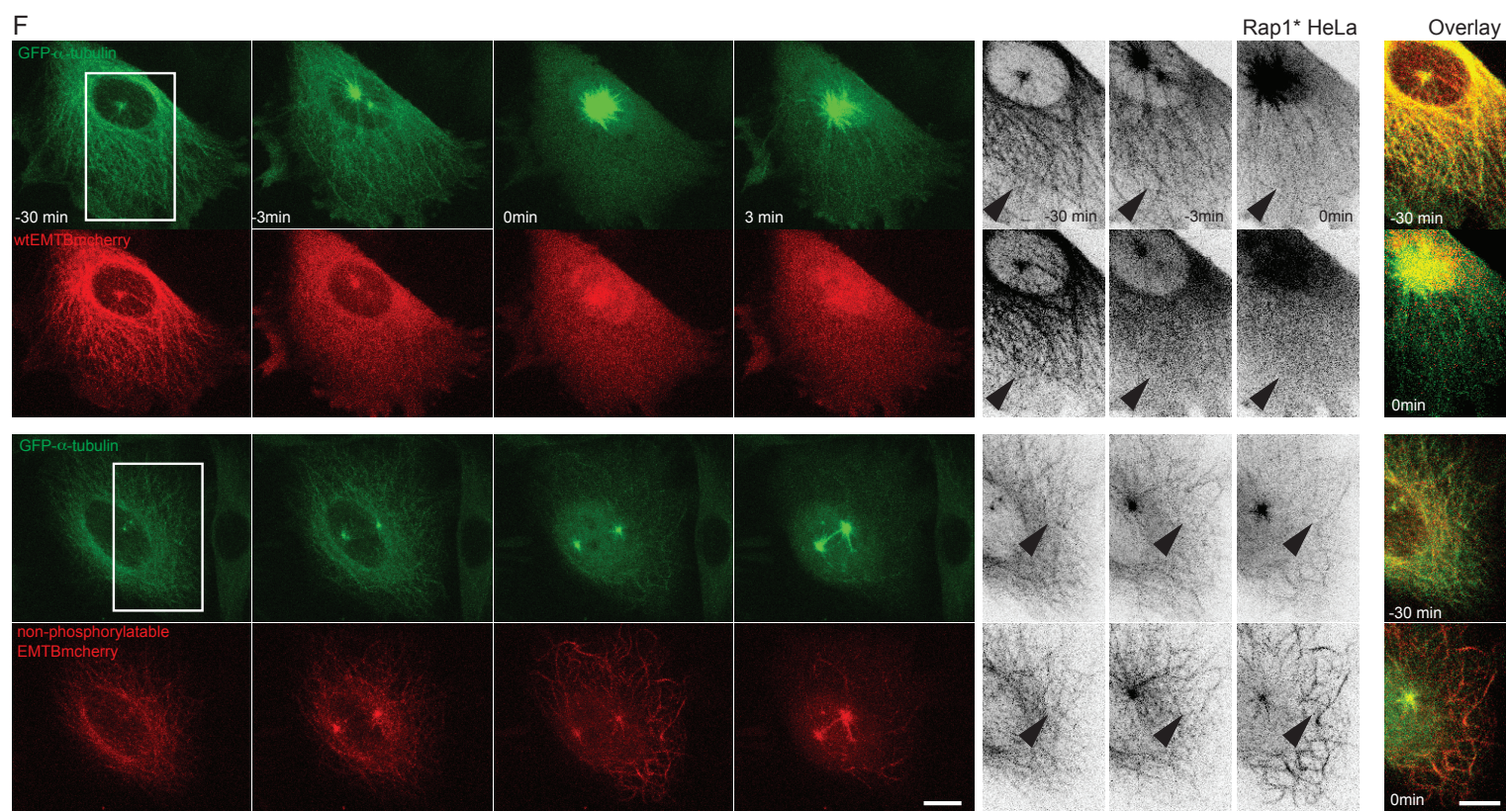

Supplement: Supplementary file 2 — Binding of Ensconsin/MAP7 to microtubules at mitotic entry is regulated by phosphorylation. A) Changes in non-centrosomal microtubule levels relative to NEP. Variance of α-tubulin-GFP signal measured as described in Additional file 1E for control siRNA (blue, five cells) and Cep192 siRNA cells (brown, five cells) for two experiments as in Fig. 2a–c. B) Western blot showing Ensconsin/MAP7 knockdown induced using three different siRNAs targeting Ensconsin/MAP7. C) Representative confocal images (x-y maximum projection) of fixed HeLa cells treated with control siRNA and three siRNAs targeting Ensconsin/MAP7. Boxed areas show regions zoomed in overlays (>10 cells per condition, one experiment). D) Representative confocal images (x-y maximum projection) of fixed MCF10A cells stained to show that Ensconsin/MAP7 is removed from microtubules in prophase compared to interphase. Ensconsin/MAP7 in red, α-tubulin in green and DAPI in blue. Boxed areas show regions zoomed in overlays, in which intensities were adjusted to remove cytoplasmic background signal (six prophase cells, > 20 interphase cells, one experiment). E) Representative confocal images of fixed HeLa cells overexpressing Rap1* in prophase stained to show that the Wt-EMTB-mCherry as well as a corresponding phospho-mimetic mutant (E-EMTB-mCherry) are largely cytoplasmic in prophase, whereas the non-phosphorylatable form (A-EMTB-mCherry) localises to the microtubules. α-Tubulin in green, Wt-EMTB-mCherry, A-EMTB-mCherry, E-EMTB-mCherry in red. Boxed areas are zoomed, shown in inverted greyscale or in overlays, where signal intensities were adjusted to remove cytoplasmic background signal (three cells per condition, one experiment). F) Representative time-lapse confocal images (x-y maximum projection) of flat (Rap1*) HeLa cells stably expressing GFP-α-tubulin and Wt-EMTB-mCherry (upper panel) or its non-phosphorylatable mutant (A-EMTB-mCherry, lower panel) to show that the non-phosphorylatable mutant stays associated w [file 12915_2017_478_MOESM2_ESM.pdf]

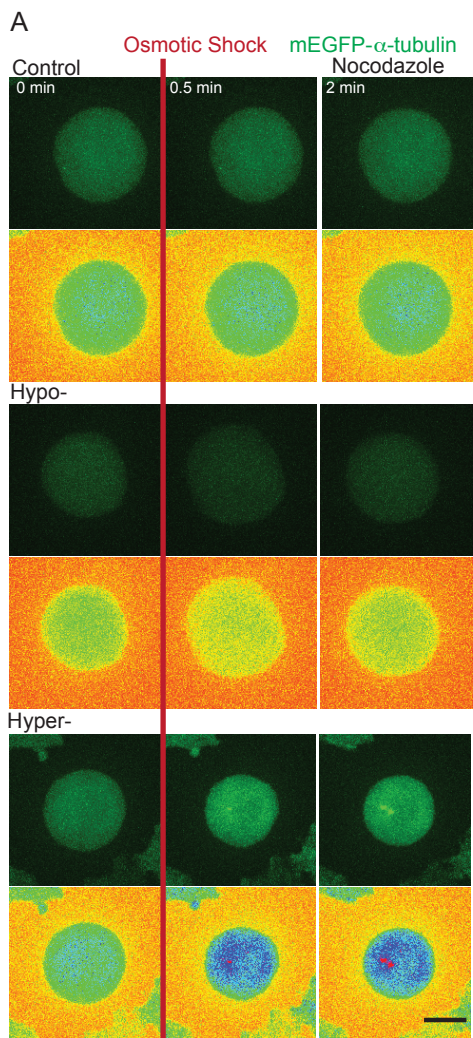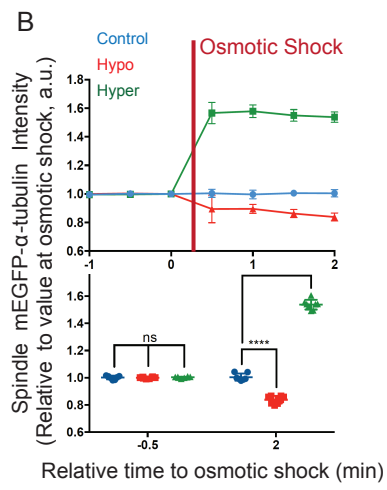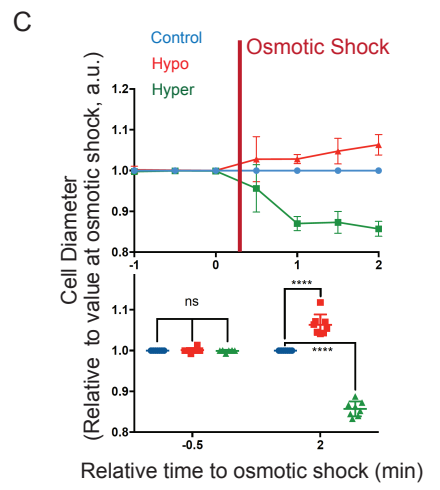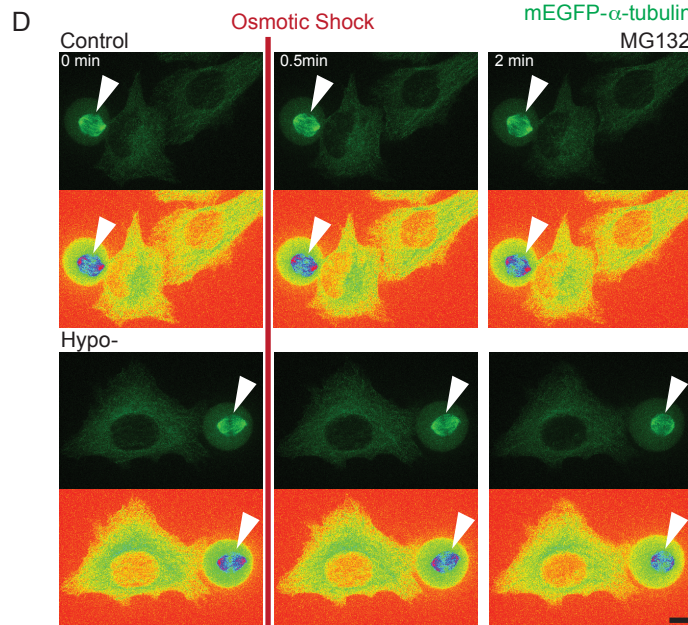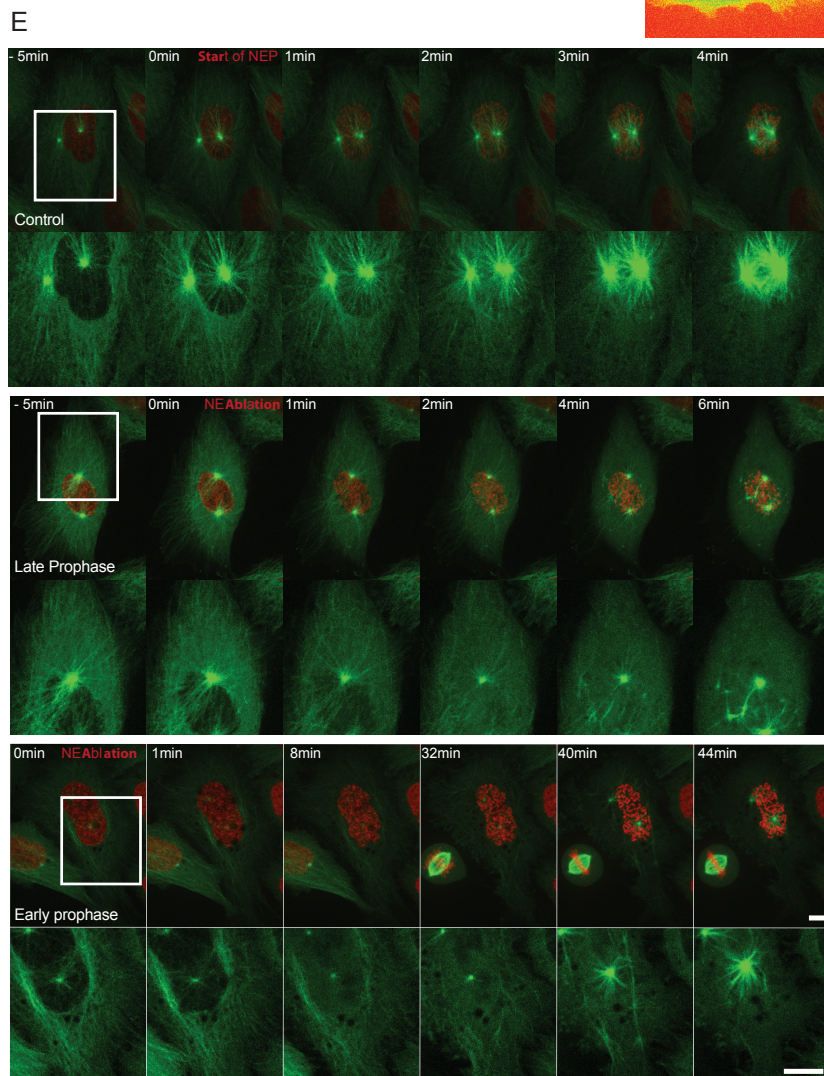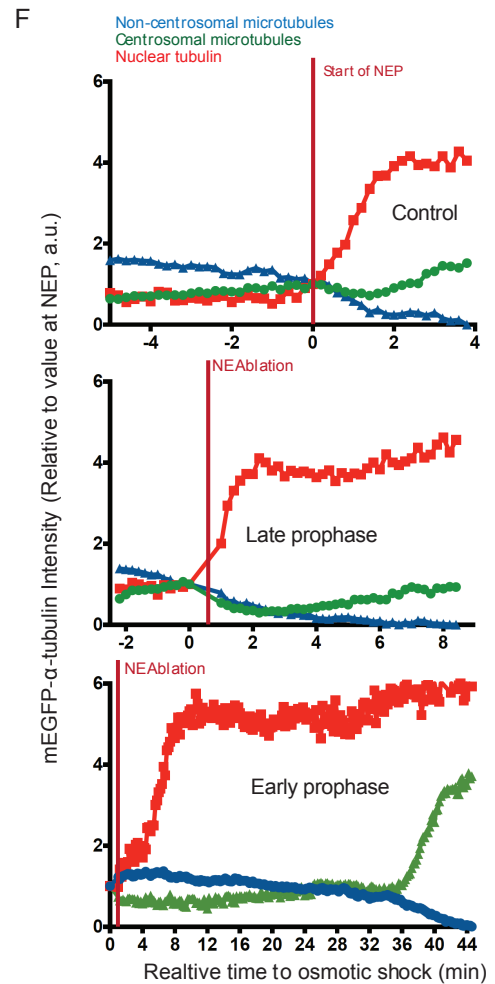

Supplement: Supplementary file 4 — Hypo-osmotic shock can be used to mimic changes in tubulin concentration induced by NEP. A) Representative time-lapse confocal images (x-y maximum projection, lower panel: pseudo-color, spectra LUT) of HeLa cells stably expressing H2B-mRFP (not imaged) and mEGFP-α-tubulin, treated with Nocodazole, to show changes in cell diameter and in mEGFP-α-tubulin intensity before and after hypo- or hyper-osmotic shock treatment relative to control treatment. Quantifications of changes in mEGFP-α-tubulin intensity (B) and in cell diameter (C) induced by osmotic shock relative to control treatment. Mean intensity of mEGFP-α-tubulin signal and cell diameter was measured in cells before and after control (blue, seven cells), hypo- (red, eight cells) or hyper- (green, eight cells) osmotic shock treatments (two independent experiments) as described in Methods. Lower panels show comparison between values at –0.5 min and 2 min relative to osmotic shock treatments. Repeated measures two-way ANOVA, Dunnett's multiple comparisons test, ****P = 0.0001. D) Representative time-lapse confocal images (x-y maximum projection, lower panel: pseudo-color, spectra LUT) of HeLa cells stably expressing H2B-mRFP (not imaged) and mEGFP-α-tubulin showing that hypo-osmotic shock affects mitotic spindle, whereas it does not have impact on interphase microtubules. White arrows indicate mitotic spindle. E) Representative time-lapse confocal images (x-y maximum projection) of HeLa cells stably expressing H2B-mRFP and mEGFP-α-tubulin and transiently overexpressing Rap1 treated with Lamin A siRNA and ESCRT-III siRNA during mitotic entry. Boxed areas are zoomed below. Control cell represents a Lamin A siRNA and ESCRT-III siRNA treated cell entering mitosis. The following cells represent accordingly a cell where nuclear envelope rupture was induced in late prophase (close to NEP) followed by immediate disassembly of microtubules and a cell where nuclear envelope rupture was induced in early prophase without tri [file 12915_2017_478_MOESM4_ESM.pdf]
